# Identifying early decline of daily function and its association with physical function in chronic kidney disease: performance-based and self-reported measures

**DOI:** 10.7717/peerj.5286

**Published:** 2018-07-18

**Authors:** Hui-Mei Chen, Shih-Ming Hsiao, Mei-Chuan Kuo, Yi-Ching Lo, Mei-Feng Huang, Yi-Chun Yeh, Cheng-Fang Yen, Cheng-Sheng Chen

**Affiliations:** 1 Department of Occupational Therapy, College of Health Science, Kaohsiung Medical University, Kaohsiung, Taiwan; 2 Graduate Institute of Medicine, College of Medicine, Kaohsiung Medical University, Kaohsiung, Taiwan; 3 Department of Nursing, Kaohsiung Medical University Hospital, Kaohsiung, Taiwan; 4 Division of Nephrology, Department of Internal Medicine, Kaohsiung Medical University Hospital, Kaohsiung, Taiwan; 5 Faculty of Renal Care, College of Medicine, Kaohsiung Medical University, Kaohsiung, Taiwan; 6 Department of Pharmacology, College of Medicine, Kaohsiung Medical University, Kaohsiung, Taiwan; 7 Graduate Institute of Natural Products, College of Pharmacy, Kaohsiung Medical University, Kaohsiung, Taiwan; 8 Department of Psychiatry, Kaohsiung Medical University Hospital, Kaohsiung, Taiwan; 9 Department of Psychiatry, College of Medicine, Kaohsiung Medical University, Kaohsiung, Taiwan

**Keywords:** Chronic kidney disease, Activities of daily living, Endurance, Muscle strength

## Abstract

**Objective:**

To verify self-reported basic and instrumental activities of daily living (IADL) with a disability and the results of performance-based tests (namely the Taiwan performance-based IADL (TPIADL), the 2-minute step test (2MST), the 30-second chair-stand test (30-s CST), and handgrip dynamometer measurement) to identify disability early and assess the associations with functional fitness in patients with advanced chronic kidney disease (CKD).

**Methods:**

A cross-sectional study of 99 patients with stage 4–5 CKD and 57 healthy elderly adults were recruited. Self-reported measures were used to collect information on basic (Barthel Index) and IADL (Lawton–Brody scale). Objective measures of the TPIADL and functional fitness (2MST, 30-s CST, handgrip dynamometer) were also assessed.

**Results:**

Only IADL, as detected by the TPIADL, were impaired to a greater extent in the CKD patients than those of healthy elderly adults. Among all the patients with CKD, a greater impairment in the TPIADL remained statistically associated with a lower ability in the 2MST. A one step increase in the 2MST score was significantly associated with an improvement of 0.2 s in the total performance time of the TPIADL.

**Conclusion:**

Performance-based measures, such as the TPIADL, may detect a functional limitation before it becomes measurable by traditional self-reported basic and IADL scales; functional limitation is mainly associated with cardiac endurance for advanced CKD.

## Introduction

Chronic kidney disease (CKD) is a common condition with a prevalence rate of 11.9%; it accounts for 0.32% of advanced-stage diseases in Taiwan (Wen et al., 2008). CKD is often associated with poor health outcomes and has become a major public health concern (Bowling et al., 2011; Jha, Wang & Wang, 2012). Disability is one of the poor outcomes of CKD (Bowling et al., 2011; Smyth et al., 2013). A multidimensional approach, including a disability assessment, has been suggested to evaluate patients with CKD (Walker et al., 2013). Disability means loss of independence in daily living activities and inability to perform tasks such as grocery shopping, housekeeping, and walking, which require certain levels of physical function (Bowling et al., 2014). Some evidence has indicated that disability may develop even in the early stages of CKD (Fried et al., 2006; Lattanzio et al., 2012; Walker et al., 2013).

Disability has been studied in depth using questionnaires related to activities of daily living (ADL), including basic activities of daily living (BADL), or instrumental activities of daily living (IADL) (Bowling et al., 2011; De Rekeneire & Volpato, 2015). Researchers of elderly populations have reported that performance-based measures of ADL function are more likely to detect early deficits in ADL function than are self-reported ADL questionnaires (Pereira et al., 2010; Rodakowski et al., 2014), particularly in the IADL domain. Therefore, performance-based measures of ADL should be optimal for early detection.

The Taiwan performance-based IADL (TPIADL) is a performance-based assessment of IADL. All skill areas in the TPIADL are central to independent community living in any population with a low educational level. A minimal level of motor skill is required to perform all the tasks. For example, an item regarding cooking required a dish’s ingredients to be named instead of assessing skills required for meal preparation. Because the assessment has an administration duration of 3–5 min and because only simple test materials are required, the task is suitable for clinical assessment in studies of large samples. It also has an established validity, indicating that the TPIADL is sensitive to impairment (Chen et al., 2015, 2016).

Furthermore, early identification of disability-associated factors is critical for interventions that are tailored for predialysis patients with CKD (Intiso, 2014). Functional fitness is the ability to perform everyday activities in a safe and independent manner without undue fatigue (Rikli & Jones, 1999). Functional fitness has been reported to be a promising measure to detect early disability (Furtado et al., 2017). Older adults with high muscle strength and cardiopulmonary endurance had a lower risk of ADL disability (Den Ouden et al., 2013; Lin et al., 2016). However, the association between IADL function and the functional fitness levels of patients with CKD still requires elaboration.

This study used separate performance-based and traditional self-reported measures to evaluate disability levels in community-dwelling predialysis patients with advanced-stage CKD. We also explored the associations of disability with functional fitness in terms of upper extremity strength, lower extremity strength, and cardiorespiratory fitness.

## Methods

### Study design and participants

This study was a case-control study at a university teaching hospital. The study protocol was approved by the Institutional Review Board of the Kaohsiung Medical University Hospital (KMUH-IRB-20120203). Written informed consent was obtained from all participants and all clinical investigations were conducted according to the principles expressed in the Declaration of Helsinki. The inclusion criteria were patients with CKD who (1) were aged ≥50 years; and (2) met the criteria for CKD according to the kidney disease outcomes quality initiative definitions, had an estimated glomerular filtration rate (eGFR) assessed using the four-variable modification of diet in renal disease study equation (Levey et al., 1999), and had advanced CKD (stage 4 or 5) with an eGFR <30 mL/min/1.73 m^2^. A non-CKD control group was recruited through an advertisement at the hospital, and the inclusion criteria were participants who (1) were aged ≥50 years; and (2) did not have a clinical history of CKD. The exclusion criteria for all participants were as follows: history of dialysis, previous renal transplantation, major neurological disorders including dementia, and severe or acute physical illness that hampered their completion of the functional fitness tests. Body mass index was calculated as weight (kilograms) divided by the square of height (meter). Physical activity was evaluated using a Chinese version of physical activity questionnaire to record the average participation time of three categories of everyday exercise. Activity was defined as level of metabolic equivalent of task (MET) and was scored and classified into three groups: <3 METs—light activity; 3–6 METs—moderate activity; and >6 METs—high activity. ICD-9-CM diagnosis codes were used to define the presence of comorbidities with CKD, such as diabetes, high blood pressure, and cardiovascular disease. The disability and functional fitness assessments were collected in an outpatient setting. One licensed and trained nurse (the second author) conducted the disability and functional fitness assessments by following a standardized procedure under the supervision of a licensed occupational therapist (the first author).

### Disability assessment

In the present study, performance-based (TPIADL) and self-reported (Lawton–Brody IADL Scale and Barthel Index (BI)) measures of ADL and physical function assessment were employed. The TPIADL (Chen et al., 2015) was designed to assess performance-based IADL in five tasks: communication, finance, cooking, shopping, and medicine use. Each task was limited to 30 s. For each task, the examiner used a digital stop watch to record the time taken to complete the task. The scoring was defined as follows: if the activity was completed accurately and within 30 s, a score of 1 was given. A score of 2 meant that the participant responded erroneously within or exceeding 30 s but corrected themselves after a verbal cue. A score of 3 meant that the participant had an erroneous response that exceeded the time limit and was not able to correct their response even when a verbal cue was given. Total scores ranged from 5 to 15, with a high score indicating low performance or capacity in IADL. Time spent completing all five tasks of the TPIADL was calculated. The Lawton–Brody IADL Scale (Lawton & Brody, 1969) is an eight-item questionnaire that assesses the following tasks: using a telephone, using public transportation, managing finances, shopping, taking medicine, food preparation, housekeeping, and washing laundry. The possible score ranges from 0 to 24; a low score is indicative of poor functioning. The self-reported BI (Mahoney & Barthel, 1965) was used to measure functional independence in basic ADL performance in this study. The BI comprises 10 BADL tasks: feeding, bathing, grooming, dressing, bowel control, bladder control, toilet use, transferring, mobility, and stair use. The possible score ranges from 0 to 100; a low score is indicative of poor functioning (Shah, Vanclay & Cooper, 1989).

### Functional fitness assessments

In this study, functional fitness was expressed through upper body strength, lower body strength, and cardiopulmonary endurance. This study used a handgrip test, a 30-second chair-stand test (30-s CST), and a 2-minute step test (2MST) as indices of functional fitness. A handgrip dynamometer (Takei scientific instruments Co., Ltd, Niigata City, Japan) was used as an indicator of upper extremity muscle strength. After explaining the procedure to the study participants and providing a demonstration, they were asked to hold the handgrip dynamometer in their dominant hands in a sitting position with their elbows flexed at 90°. Two consecutive measurements were performed for assessment. The mean of the maximal values for each hand was used for analysis (Hiraki et al., 2013). The handgrip muscle strength was recorded in kilograms, as indicated by the pointer on the dynamometer. The 30-s CST (Jones, Rikli & Beam, 1999) assessed the number of sit-to-stand repetitions completed in 30 s and served as a proxy indicator of lower-limb muscle strength. A standard chair without arm rests (height 43 cm) was used. The 2MST measured cardiorespiratory endurance and was scored by quantifying the number of full steps completed in 2 min. For each step, the participants were required to raise each knee to the required stepping height. A mark was placed on a wall at a height corresponding to the midpoint between the participants’ standing patella and iliac crest height (Chang et al., 2015), with the participants needing to raise their knee to this height for each step to be counted. The score was the number of successful right knee raises at the required height. The order of test administration was not predetermined, and was based on equipment and tester availability. Participants’ exercise capacity was also evaluated using a questionnaire to record exercise form and duration.

### Data analysis

Descriptive analyses were completed and presented as means and standard deviations for continuous data and as percentages for categorical data. A Chi-squared test was used to compare sex and comorbidity differences between participants with and without CKD, and a *t*-test was used to examine differences in their age and education level. Group comparison for the TPIADL, Lawton–Brody IADL, BI, 2MST, handgrip and 30-s CST scores was performed using analysis of covariance (ANCOVA) to adjust for age, sex, education duration. Because the distribution of the TPIADL scores of the participants with CKD was skewed, the TPIADL was dichotomized as with disability (scored as “>5”) or without disability (scored as “5”) for clinical correlation analyses. General linear modeling was used to determine the associations between disability parameters (dependent variables) and functional fitness tests (independent variables), with adjustments for age, sex, and physical activity (yes/no exercise). All statistical tests were two-sided, and results were considered significant at *p* < 0.05. Statistical analyses were conducted using SPSS 18.0 for Windows (SPSS Inc., Chicago, IL, USA).

## Results

### Participants’ characteristics

Table 1 presents comparisons of demographic and clinical data, performance-based and self-reported ADL measurements, and functional fitness test results among the 99 participants with CKD (50 stage 4, 49 stage 5) and among the 57 non-CKD subjects (the non-CKD group). No significant differences in age (*p* = 0.36) or sex (*p* = 0.31) were discovered between the CKD and non-CKD groups. The CKD group had a lower education duration (*p* < 0.01) and exercise capacity than the non-CKD group (*p* < 0.01). The CKD group had a higher rate of diabetes (*p* < 0.01), high blood pressure (*p* < 0.01), and hyperlipidemia (*p* < 0.01) than the non-CKD group, but no significant differences existed in the prevalence of cardiovascular disease (*p* = 0.25) and osteoarthritis (*p* = 0.57) between the CKD and non-CKD groups. The CKD group had a higher rate of overall comorbidity than the non-CKD group.

**Table 1:** Demographic and clinical characteristics of participants with and without CKD.

	CKD (*n* = 99)	Non-CKD (*n* = 57)	*t* or χ^2^ or *F*	*p*-Value
**Demographic variables**				
Age (years), mean (SD)	67.0 (7.8)	65.8 (6.5)	−0.92	0.36^a^
Sex (male), *n* (%)	50 (51)	24 (42)	1.02	0.31^b^
Education (years), mean (SD)	8.8 (4.4)	11.0 (4.0)	3.09	<0.01^a^
Body mass index (kg/m^2^), mean (SD)	24.2 (3.9)	23.5 (3.4)	−1.17	0.26^a^
**Physical activity, *n* (%)**				
No exercise	8 (8.1)	2 (3.5)	13.63	<0.01^b^
Low	72 (72.7)	31 (54.4)		
Middle	19 (19.2)	20 (35.1)		
High	0	4 (7)		
**Comorbidities, *n* (%)**				
Diabetes	44 (44.4)	5 (8.8)	21.37	<0.01^b^
Hypertension	79 (79.8)	18 (31.6)	35.76	<0.01^b^
Cardiovascular disease	19 (19.2)	7 (12.3)	1.31	0.25^b^
Osteoarthritis	2 (2.0)	2 (3.6)	0.32	0.57^b^
**Daily function, mean (SD)**				
TPIADL				
Total score	5.34 (0.65)	5.05 (0.23)	5.56	0.02^c^
Time	51.33 (20.0)	39.0 (16.0)	7.72	0.006^c^
IADL	23.4 (1.2)	23.5 (0.7)	1.49	0.27^c^
BI	99.7 (2.0)	100 (0)	0.84	0.38^c^
**Functional fitness, mean (SD)**				
Grip strength	25.9 (7.8)	27.43 (8.4)	5.00	0.03^c^
30-s chair-stand test	10.8 (2.9)	11.88 (3.7)	3.54	0.06^c^
2-min step test	102.0 (18.5)	117.3 (33.6)	13.37	<0.01^c^

**Notes:**

CKD, chronic kidney disease; non-CKD, normal; BI, Barthel Index; IADL, Lawton–Brody IADL scale; SD, standard deviation; TPIADL, Taiwan performance-based IADL.

a *t*-test.

b Chi-square test.

c ANCOVA test.

### Comparison of performance-based and self-reported IADL

The ANCOVA results revealed that after adjusting for age, sex, and educational level, the CKD group had higher total scores (*p* = 0.02) and longer times (*p* = 0.03) in the TPIADL than the non-CKD group, indicating that the CKD group had poorer performance-based IADL than the non-CKD group. However, no significant differences existed in the self-reported Lawton–Brody IADL Scale (*p* = 0.22) and BI (*p* = 0.36) scores between the CKD and non-CKD groups.

### Comparison of functional fitness

After adjusting for age and sex, the CKD group had lower handgrip strength and lower 2MST scores than did the non-CKD group (*p* < 0.01 and *p* = 0.03, respectively). No significant difference existed in the 30-s CST score between the CKD and non-CKD groups (*p* = 0.06).

### Association between the TPIADL and functional fitness

Table 2 presents a comparison of functional fitness between the patients with CKD with (*n* = 74) and without (*n* = 25) a TPIADL-measured disability. After adjusting for age, sex, and physical activity, the patients with CKD with a TPIADL-measured disability had a lower 2MST than did those without a disability (with disability: 95.0 ± 24.4; without disability: 104.2 ± 15.8, *p* < 0.05). However, no differences existed in the handgrip dynamometer score (with disability: 21.8 ± 7.5; without disability: 27.2 ± 7.5, *p* = 0.10) or the 30-s CST score (with disability: 10.9 ± 2.9; without disability: 10.6 ± 2.9, *p* = 0.76) between the patients with CKD with and without TPIADL-measured disability. The results only indicated that the TPIADL result was correlated with cardiorespiratory endurance capacity. In patients with CKD, performance-based IADL impairment was significantly associated with a low level of aerobic endurance, but not with grip strength or lower extremity strength.

**Table 2:** Comparison of functional fitness in participants with CKD with and without TPIADL disability.

Functional fitness	TPIADL = 5 (*n* = 74)	TPIADL > 5 (*n* = 25)	*F*	*p*-Value
Grip strength	27.2 (7.5)	21.8 (7.5)	2.71	0.10
30-s chair-stand test	10.9 (2.9)	10.6 (2.9)	0.09	0.76
2-min step test	104 (15.8)	95 (24.4)	4.40	0.04

**Note:**

CKD, chronic kidney disease; TPIADL, Taiwan performance-based IADL.

The TPIADL completion time appeared to exhibit normal stem and leaf distribution. Multiple linear regression was used to further investigate the association of functional fitness indices and physical activity with TPIADL completion time, after adjustments had been made for age and education. In the CKD group, a relatively long TPIADL completion time was associated with a relatively low 2MST score (*R*^2^ = 0.46, *F* = 12.719, *p* < 0.001 for the model) (Table 3). The one-step increase in the 2MST score was significantly associated with an improvement of 0.2 s in total performance time of TPIADL. However, we found that handgrip (*p* = 0.397), 30-s CST (*p* = 0.298) and current physical activity (*p* = 0.616) were not associated with the total performance time of TPIADL.

**Table 3:** Linear regression analysis of the association of functional fitness and physical activity with TPIADL time score adjustment for age and education years in patients with chronic kidney disease.

	Unstandardized regression coefficient (B)	Standardized regression coefficient (β)	*t*-Value	*p*-Value
Grip strength	−0.22	−0.086	−0.851	0.397
30-s chair stand test	0.60	0.087	1.047	0.298
2-min step test	−0.18	−0.171	−2.145	0.035
Physical activity	−2.76	−0.039	−0.503	0.616

**Notes:**

Longer TPIADL complete time was associated with worse 2-min step score.

TPIADL, Taiwan performance-based IADL.

Adjusted *R*^2^ = 0.46.

## Discussion

Our study revealed that, compared with individuals without CKD, patients with advanced CKD had compromised IADL levels as assessed by the TPIADL and poorer functional fitness in terms of upper extremity strength and cardiorespiratory fitness. Poorer performance-based IADL levels in patients with advanced CKD were associated with a lower cardiorespiratory endurance capacity, but were not associated with grip strength or lower extremity strength after adjusting for covariates. An increase in cardiovascular capacity correlated with accelerated completion time of performance-based IADL in patients with advanced CKD.

Unlike conventional self-reported IADL assessment, performance-based IADL assessment was able to detect mild IADL impairment in patients with CKD in this study. Individuals with advanced CKD may present increasing limitations in the performance of complex everyday activities, although they are still able to do them mostly independently. In this study, the poorer TPIADL performance of the CKD group was reflected in their low overall task accuracy and the long times they required for task completion. Therefore, measuring the complete time of an IADL task is vital to early detection of daily living impairment. The results also implied that using objective measures may be more sensitive to identify early signs of everyday disability in patients with advanced CKD than self-rated measures. Patients with advanced CKD experienced difficulties in completing IADL tasks when the task required efficient performance, suggesting that performance-based measures offer a superior discriminative capacity over self-reported function measures in patients with advanced CKD. The TPIADL is a potentially useful tool for screening early stages of disability among patients with advanced CKD.

Evidence suggested that using objective measures may help identify early signs of physical function impairment (Brach et al., 2002). Our data supported that patients with advanced CKD (eGFR level <30 mL/min/1.73 m^2^) were more likely to have poorer cardiorespiratory endurance and less strength in the upper extremity than those without CKD, even though they were self-rated as near-independent using BADL or IADL scales. These results were consistent with previous studies that have demonstrated significant impairment in some aspects of functional fitness in predialysis patients with CKD (Chang et al., 2011; Hiraki et al., 2013; Roshanravan et al., 2013; Walker et al., 2013). Patients with cardiovascular limitations may require additional effort or time to complete an IADL task and an efficient cardiorespiratory endurance capacity may be crucial for the prevention of early IADL disability. A study identified lower peak oxygen consumption values in patients with CKD stages 3–5 (Nelson et al., 2016), which suggests numerous patients with CKD who have poor cardiovascular fitness may experience difficulty efficiently completing time-sensitive IADL tasks, such as crossing roads or driving in heavy traffic; that study indicated that decreased cardiovascular endurance observed in patients with CKD may be an early warning sign of a future need for assistance in specific IADL tasks. At this early stage of decline, interventions to promote cardiovascular functional fitness may be a potentially cost-effective approach. Another study reported that patients with CKD who have regular exercise habits may preserve their cardiorespiratory endurance better than those who do not (Stump, 2011). We suggest that patients with advanced CKD may require a behavioral intervention to promote healthy lifestyle habits for improving their cardiorespiratory endurance, which may in turn prevent the early onset of disability.

However, muscle strength index was not associated with performance-based IADL completion time. This implied that fast completion of cognitive-skill IADL tasks may not depend on a high level of muscle strength in the upper or lower extremity. That is, patients with advanced CKD may spend a “normal” amount of time on a task, but they are more likely to experience considerable shortness of breath or a fast heart rate, rather than feel muscle stress in their limbs. In this study, the TPIADL with a minimal motor requirement could be considered as a processing-speed based measure. Similar reports indicated patients with a longer CKD duration had an increased probability of relatively poor performance on processing-speed tests of cognitive ability (Owolabi et al., 2016). Cardiovascular risk factors may have adverse effects on the cerebral health of patients with CKD. Future studies are required to determine what type of exercise training programs may best improve processing speed of IADL performance in patients of long CKD duration.

A strength of our study was that it was specifically designed to assess CKD-associated functional decline by examining concurrent performance-based and self-reported measures. Our findings provided empirical support for the need to employ more sensitive and objective measures of everyday function to improve the clinical care of patients with CKD. These objective measures for IADL functioning should assess task accuracy and task completion time. The results of this study also indicated a need for future studies to determine whether recognizing and intervening in functional impairment can alter the trajectory of functional change.

This study had some limitations. First, patients who were unable to independently ambulate and undergo the functional fitness tests were excluded. The results therefore cannot be generalized to all patients with CKD. Second, debate exists regarding whether 30-s CSTs are affected by multiple physiological and psychological factors, and therefore 30-s CSTs cannot serve as a proxy measure of lower-limb strength among older people (Lord et al., 2002). In this study, we did not measure physical and psychological variables that can be adjusted in statistical analyses. Third, the measures for functional fitness in this study were easily conducted but may not be sensitive enough to determine the effects of aerobic fitness. For instance, maximum oxygen uptake from a peak-exercise test can provide information on the maximum level of integrated functioning in cardiopulmonary and neuromuscular systems (Painter & Marcus, 2013). Comprehensive muscle strength requires assessment through maximum voluntary contraction or even one repetition maximum of large muscle groups (Zanini et al., 2015). Fourth, to minimize confounding effects resulting from fixed orders, we did not follow a fixed order to assess functional fitness; nevertheless, a convenience order may also lead to certain confounding effects. Therefore, adopting a randomized administration sequence or sufficient rest interval between tests in future studies is advisable. Fifth, a ceiling effect may exist in the TPIADL for most patients with advanced CKD, which suggests that a minor change in IADL performance may not always be detectable using the TPIADL. Sixth, although our results did not reveal physical activity status of patients to be associated with the total performance time of TPIADL, a type II error caused by the small sample size was still possible. Finally, the cross-sectional design of this study limited the ability to determine causal relationships between functional fitness impairment and IADL disability.

## Conclusion

This study provided evidence that the objective measure of the TPIADL could be used to identify mild deficits in daily function among elderly patients with advanced CKD. Functional fitness in terms of cardiorespiratory capacity was revealed to be associated with IADL disability in this population. These findings provide potential targets for intervention to prevent the onset of disability.

## Supplemental Information

10.7717/peerj.5286/supp-1 Supplemental Information 1 Raw data in CKD study.

10.7717/peerj.5286/supp-2 Supplemental Information 2 Evaluation tool in CKD study.

10.7717/peerj.5286/supp-3 Supplemental Information 3 Evaluation tool for disability. List of disability scale (Lawton scale & Barthel Index) or tool (TPIADL).

## Additional Information and Declarations

### Data Availability

The following information was supplied regarding data availability: The raw data are provided in a Supplemental File.

## References

[ref-1] Bowling CB, Muntner P, Sawyer P, Sanders PW, Kutner N, Kennedy R, Allman RM (2014). Community mobility among older adults with reduced kidney function: a study of life-space. American Journal of Kidney Diseases.

[ref-2] Bowling CB, Sawyer P, Campbell RC, Ahmed A, Allman RM (2011). Impact of chronic kidney disease on activities of daily living in community-dwelling older adults. Journals of Gerontology Series A: Biological Sciences and Medical Sciences.

[ref-3] Brach JS, VanSwearingen JM, Newman AB, Kriska AM (2002). Identifying early decline of physical function in community-dwelling older women: performance-based and self-report measures. Physical Therapy.

[ref-4] Chang KV, Hung CY, Li CM, Lin YH, Wang TG, Tsai KS, Han DS (2015). Reduced flexibility associated with metabolic syndrome in community-dwelling elders. PLOS ONE.

[ref-5] Chang YT, Wu HL, Guo HR, Cheng YY, Tseng CC, Wang MC, Lin CY, Sung JM (2011). Handgrip strength is an independent predictor of renal outcomes in patients with chronic kidney diseases. Nephrology Dialysis Transplantation.

[ref-6] Chen HM, Lin HF, Huang MF, Chang CW, Yeh YC, Lo YC, Yen CF, Chen CS (2016). Validation of Taiwan performance-based instrumental activities of daily living (TPIADL), a performance-based measurement of instrumental activities of daily living for patients with vascular cognitive impairment. PLOS ONE.

[ref-7] Chen HM, Yeh YC, Su WL, Huang MF, Chang CW, Chen CS (2015). Development and validation of a new performance-based measurement of instrumental activities of daily living in Taiwan. Psychogeriatrics.

[ref-8] De Rekeneire N, Volpato S (2015). Physical function and disability in older adults with diabetes. Clinics in Geriatric Medicine.

[ref-9] Den Ouden MEM, Schuurmans MJ, Brand JS, Arts IEMA, Mueller-Schotte S, van der Schouw YT (2013). Physical functioning is related to both an impaired physical ability and ADL disability: a ten year follow-up study in middle-aged and older persons. Maturitas.

[ref-10] Fried LF, Lee JS, Shlipak M, Chertow GM, Green C, Ding J, Harris T, Newman AB (2006). Chronic kidney disease and functional limitation in older people: health, aging and body composition study. Journal of the American Geriatrics Society.

[ref-11] Furtado G, Patricio M, Loureiro M, Teixeira AM, Ferreira JP (2017). Physical fitness and frailty syndrome in institutionalized older women. Perceptual and Motor Skills.

[ref-12] Hiraki K, Yasuda T, Hotta C, Izawa KP, Morio Y, Watanabe S, Sakurada T, Shibagaki Y, Kimura K (2013). Decreased physical function in pre-dialysis patients with chronic kidney disease. Clinical and Experimental Nephrology.

[ref-13] Intiso D (2014). The rehabilitation role in chronic kidney and end stage renal disease. Kidney and Blood Pressure Research.

[ref-14] Jha V, Wang AYM, Wang H (2012). The impact of CKD identification in large countries: the burden of illness. Nephrology Dialysis Transplantation.

[ref-15] Jones CJ, Rikli RE, Beam WC (1999). A 30-s chair-stand test as a measure of lower body strength in community-residing older adults. Research Quarterly for Exercise and Sport.

[ref-16] Lattanzio F, Corsonello A, Abbatecola AM, Volpato S, Pedone C, Pranno L, Laino I, Garasto S, Corica F, Passarino G, Antonelli Incalzi R (2012). Relationship between renal function and physical performance in elderly hospitalized patients. Rejuvenation Research.

[ref-17] Lawton MP, Brody EM (1969). Assessment of older people: self-maintaining and instrumental activities of daily living. Gerontologist.

[ref-18] Levey AS, Bosch JP, Lewis JB, Greene T, Rogers N, Roth D (1999). A more accurate method to estimate glomerular filtration rate from serum creatinine: a new prediction equation. Modification of Diet in Renal Disease Study Group. Annals of Internal Medicine.

[ref-19] Lin PS, Hsieh CC, Cheng HS, Tseng TJ, Su SC (2016). Association between physical fitness and successful aging in Taiwanese older adults. PLOS ONE.

[ref-20] Lord SR, Murray SM, Chapman K, Munro B, Tiedemann A (2002). Sit-to-stand performance depends on sensation, speed, balance, and psychological status in addition to strength in older people. Journals of Gerontology Series A: Biological Sciences and Medical Sciences.

[ref-21] Mahoney FI, Barthel DW (1965). Functional Evaluation: The Barthel Index. Maryland State Medical Journal.

[ref-22] Nelson A, Otto J, Whittle J, Stephens RCM, Martin DS, Prowle JR, Ackland GL (2016). Subclinical cardiopulmonary dysfunction in stage 3 chronic kidney disease. Open Heart.

[ref-23] Owolabi LF, Abdu A, Ibrahim A, Owolabi DS, Nalado A, Bappa A, Taura AA (2016). Related factors and predictors of cognitive dysfunction in chronic kidney disease on maintenance hemodialysis in Nigeria. Journal of Neurosciences in Rural Practice.

[ref-24] Painter P, Marcus RL (2013). Assessing physical function and physical activity in patients with CKD. Clinical Journal of the American Society of Nephrology.

[ref-25] Pereira FS, Yassuda MS, Oliveira AM, Diniz BS, Radanovic M, Talib LL, Gattaz WF, Forlenza OV (2010). Profiles of functional deficits in mild cognitive impairment and dementia: benefits from objective measurement. Journal of the International Neuropsychological Society.

[ref-26] Rikli RE, Jones CJ (1999). Development and validation of a functional fitness test for community-residing older adults. Journal of Aging and Physical Activity.

[ref-27] Rodakowski J, Skidmore ER, Reynolds CF, Dew MA, Butters MA, Holm MB, Lopez OL, Rogers JC (2014). Can performance on daily activities discriminate between older adults with normal cognitive function and those with mild cognitive impairment?. Journal of the American Geriatrics Society.

[ref-28] Roshanravan B, Robinson-Cohen C, Patel KV, Ayers E, Littman AJ, de Boer IH, Ikizler TA, Himmelfarb J, Katzel LI, Kestenbaum B, Seliger S (2013). Association between physical performance and all-cause mortality in CKD. Journal of the American Society of Nephrology.

[ref-29] Shah S, Vanclay F, Cooper B (1989). Improving the sensitivity of the Barthel Index for stroke rehabilitation. Journal of Clinical Epidemiology.

[ref-30] Smyth A, Glynn LG, Murphy AW, Mulqueen J, Canavan M, Reddan DN, O’Donnell M (2013). Mild chronic kidney disease and functional impairment in community-dwelling older adults. Age and Ageing.

[ref-31] Stump CS (2011). Physical activity in the prevention of chronic kidney disease. Cardiorenal Medicine.

[ref-32] Walker SR, Gill K, Macdonald K, Komenda P, Rigatto C, Sood MM, Bohm CJ, Storsley LJ, Tangri N (2013). Association of frailty and physical function in patients with non-dialysis CKD: a systematic review. BMC Nephrology.

[ref-33] Wen CP, Cheng TYD, Tsai MK, Chang YC, Chan HT, Tsai SP, Chiang PH, Hsu CC, Sung PK, Hsu YH, Wen SF (2008). All-cause mortality attributable to chronic kidney disease: a prospective cohort study based on 462 293 adults in Taiwan. Lancet.

[ref-34] Zanini A, Aiello M, Cherubino F, Zampogna E, Azzola A, Chetta A, Spanevello A (2015). The one repetition maximum test and the sit-to-stand test in the assessment of a specific pulmonary rehabilitation program on peripheral muscle strength in COPD patients. International Journal of Chronic Obstructive Pulmonary Disease.

