# Supplementary material for: Identifying early decline of daily function and its association with physical function in chronic kidney disease: performance-based and self-reported measures
Source: PeerJ. 2018 Jul 18;6:e5286. doi: 10.7717/peerj.5286 (PMC6054786; doi:10.7717/peerj.5286)
Supplement: Supplemental Information 2 [file peerj-06-5286-s002.docx]

**工具性日常生活活動能力量表（Lawton IADL）**

| 1. **上街購物 【□ 不適用 (勾選“不適用”者，此項分數視為滿分)】**   □3.獨立完成所有購物需求  □2.獨立購買日常生活用品  □1.每一次上街購物都需要有人陪  □0.完全不會上街購物 | |  |
| --- | --- | --- |
| 1. **外出活動 【□ 不適用 (勾選“不適用”者，此項分數視為滿分)】**   □4.能夠自己開車、騎車  □3.能夠自己搭乘大眾運輸工具  □2.能夠自己搭乘計程車但不會搭乘大眾運輸工具  □1.當有人陪同可搭計程車或大眾運輸工具  □0.完全不能出門 | |  |
| 1. **食物烹調 【□ 不適用 (勾選“不適用”者，此項分數視為滿分)】**   □3.能獨立計畫、烹煮和擺設一頓適當的飯菜  □2.如果準備好一切佐料，會做一頓適當的飯菜  □1.會將已做好的飯菜加熱  □0.需要別人把飯菜煮好、擺好 | |  |
| 1. **家務維持 【□ 不適用 (勾選“不適用”者，此項分數視為滿分)】**   □4.能做較繁重的家事或需偶爾家事協助（如搬動沙發、擦地板、洗窗戶）  □3.能做較簡單的家事，如洗碗、鋪床、疊被  □2.能做家事，但不能達到可被接受的整潔程度  □1.所有的家事都需要別人協助  □0.完全不會做家事 | |  |
| 1. **洗衣服 【□ 不適用 (勾選“不適用”者，此項分數視為滿分)】**   □2.自己清洗所有衣物  □1.只清洗小件衣物  □0.完全依賴他人 | |  |
| 1. **使用電話的能力 【□ 不適用 (勾選“不適用”者，此項分數視為滿分)】**   □3.獨立使用電話，含查電話簿、撥號等  □2.僅可撥熟悉的電話號碼  □1.僅會接電話，不會撥電話  □0.完全不會使用電話 | |  |
| **7.服用藥物 【□ 不適用 (勾選“不適用”者，此項分數視為滿分)】**  □3.能自己負責在正確的時間用正確的藥物  □2.需要提醒或少許協助  □1.如果事先準備好服用的藥物份量，可自行服用  □0.不能自己服用藥物 | |  |
| **8.處理財務能力【□ 不適用 (勾選“不適用”者，此項分數視為滿分)】**  □2.可以獨立處理財務  □1.可以處理日常的購買，但需要別人協助與銀行往來或大宗  買賣  □0.不能處理錢財 | |  |
| **總分** |  | |

**巴氏量表(Barthel Index)**

| **項 目** | **分數** | 內 容 說 明 |
| --- | --- | --- |
| 1.進食 | 10□  5□  0□ | 自己在合理時間(約十秒鐘吃一口)可用筷子取食眼前的食物。若需進食輔具時，應會自行穿脫。  需別人幫忙穿脫輔具或只會用湯匙進食。  無法自行取食或耗費時間過長。 |
| 2.個人衛生 | 5□  0□ | 可以自行洗手、刷牙、洗臉及梳頭。  需要他人部份或完全協助。 |
| 3.上廁所 | 10□  5□  0□ | 可自行上下馬桶、穿脫衣服、不弄髒衣服、會自行使用衛生紙擦拭。  需要協助保持姿勢的平衡、整理衣服或用衛生紙。  無法自己完成。 |
| 4.洗澡 | 5□  0□ | 能獨立完成(不論是盆浴或沐浴)，不需別人在旁。  需別人協助。 |
| 5.穿脫衣服 | 10□  5□  0□ | 能自己穿脫衣服、鞋子，自己扣釦子、上拉鍊或綁鞋帶。  在別人協助下，可自己完成一半以上的動作。  不會自己做。 |
| 6.大便控制 | 10□  5□  0□ | 不會失禁，能自行灌腸或使用塞劑。  偶爾會失禁(每週不超過一次)，需要他人協助使用灌腸或塞劑。  失禁，無法自己控制且需他人處理。 |
| 7.小便控制 | 10□  5□  0□ | 能自己控制不會有失禁，或能自行使用並清潔尿套、尿袋。  偶爾會失禁(每週不超過一次)或尿急(無法等待放好變盆或及時趕到廁所)或需要他人協助處理尿套。  失禁，無法自己控制且需他人處理。 |
| 8.平地行走 | 15□  10□  5□  0□ | 使用或不使用輔具，皆可獨立行走50公尺以上。  需他人稍微扶持或口頭指導才能行走50公尺以上。  雖無法行走，但可以操作輪椅(包括轉彎、進門及接近桌子、床沿)並可推行輪椅50公尺以上。  完全無法自行行走，需別人幫忙推輪椅。 |
| 9.上下樓梯 | 10□  5□  0□ | 可自行上下樓梯，可使用扶手、柺杖等輔具。  需他人協助或監督才能上下樓梯。  無法上下樓梯。 |
| 10.上下床或椅子 | 15□  10□  5□  0□ | 整個過程可獨立完成。  移動身體時需要稍微協助、給予提醒、安全監督。  可以自行坐起，但從床上坐起時或移動身體時需要他人協助。  不會自己移動。 |
| **總分** |  | |

**TPIADL form**

| **活動名稱** | **題目** | **完成**  **時間** | **時間**  **限制** | **表現計分** |
| --- | --- | --- | --- | --- |
| 一、電話簿 | **肉粽店** |  | 30  seconds | 練習題 |
|  | **加油站** |  |  | 練習題 |
|  | **牙科診所** |  |  | 🞎 (1)  🞎 (2)  🞎 (3) |
| 二、硬幣換算 | **67元** |  |  | 🞎 (1)  🞎 (2)  🞎 (3) |
| 三、食物的營養配方 | **漢堡** |  |  | 🞎 (1)  🞎 (2)  🞎 (3) |
| 四、找出物品 | **蘆筍汁與脆瓜罐頭** |  |  | 🞎 (1)  🞎 (2)  🞎 (3) |
| 五、藥物的使用方式  ※右下角編有頁碼依順序施測 | **藥袋1** |  |  | 練習題 |
|  | **藥袋2** |  |  | 🞎 (1)  🞎 (2)  🞎 (3) |
|  | **總時間** |  | **總分** |  |
