# Supplementary material for: Identifying early decline of daily function and its association with physical function in chronic kidney disease: performance-based and self-reported measures
Source: PeerJ. 2018 Jul 18;6:e5286. doi: 10.7717/peerj.5286 (PMC6054786; doi:10.7717/peerj.5286)
Supplement: Supplemental Information 3 — List of disability scale (Lawton scale & Barthel Index) or tool (TPIADL). [file peerj-06-5286-s003.pdf]

Lawton IADL Scale

**Item and description**	**score**
**1. Shopping**<br>□ Takes care of all shopping needs independently...3<br>□ Shops independently for small purchases ....2<br>□ Needs to be accompanied on any shopping trip ....1<br>□ Completely unable to shop 0	
**2.** Using public transportation<br>□ Travels independently on public transportation or drives own car ....4<br>□ Arranges own travel via taxi, but does not otherwise use public transportation..........3<br>□ Travels on public transportation when assisted or accompanied by another................2<br>□ Travel limited to taxi or automobile with assistance of another ....1<br>□ Does not travel at all 0	
**3. Food preparation**<br>□ Plans, prepares, and serves adequate meals independently..3<br>□ Prepares adequate meals if supplied with ingredients..2<br>□ Heats and serves prepared meals or prepares meals but does not maintain adequate diet ....1<br>□ Needs to have meals prepared and served 0	
**4.** Housekeeping<br>□ Maintains house alone with occasion assistance (heavy work)...................................4<br>□ Performs light daily tasks such as dishwashing, bed making…...................................3<br>□ Performs light daily tasks, but cannot maintain acceptable level of cleanliness ....2<br>□ Needs help with all home maintenance tasks ....1<br>□ Does not participate in any housekeeping tasks...0	
**5. Washing laundry**<br>□ Does personal laundry completely...2<br>□ Launders small items, rinses socks, stockings, etc ....1<br>□ All laundry must be done by others 0	
6.**Using the telephone**<br>□ Operates telephone on own initiative; looks up and dials numbers.............................3<br>□ Dials a few well-known numbers...2<br>□ Answers telephone, but does not dial...1<br>□ Does not use telephone at all..0	
**7. Taking medicine**<br>□ Is responsible for taking medication in correct dosages at correct time ....2<br>□ Takes responsibility if medication is prepared in advance in separate dosages...........1<br>□ Is not capable of dispensing own medication..0	

**8. Managing finances**<br>□ Manages financial matters independently (budgets, writes checks, pays rent and bills, goes to bank); collects and keeps track of income……………………………..…2<br>□ Manages day-to-day purchases, but needs help with banking, major purchases, etc ....1<br>□ Incapable of handling money 0	
**Total score**	

Barthel Index

Item	Score & Description
**1. Feeding**	0 = unable<br>5 = needs help cutting, spreading butter, etc.<br>10 = independent (food provided within reach)
**2. Grooming**	0 = needs help with personal care<br>5 = independent face/hair/teeth/shaving (implements provided)
**3. Toilet use**	0 = dependent<br>5 = needs some help, but can do something alone<br>10 = independent (on and off, dressing, wiping)
**4. Bathing**	0 = dependent<br>5 = independent (or in shower)
**5. Dressing**	0 = dependent<br>5 = needs help, but can do about half unaided<br>10 = independent (including buttons, zips, laces, etc.)
**6. Bowels**	0 = incontinent (or needs to be given enemata)<br>5 = occasional accident (once/week)<br>10= continent
**7. Bladder**	0 = incontinent, or catheterized and unable to manage<br>5 = occasional accident (max. once per 24 hours)<br>10 = continent (for over 7 days)
**8. Mobility**	0 = immobile<br>5 = wheelchair independent, including corners, etc.<br>10 = walks with help of one person (verbal or physical)<br>15 = independent (but may use any aid, e.g., stick)
**9. Stairs**	0 = unable<br>5 = needs help (verbal, physical, carrying aid)<br>10 = independent up and down
**10. Transfer**	0 = unable – no sitting balance<br>5 = major help (one or two people, physical), can sit<br>10 = minor help (verbal or physical)<br>15 = independent
Total score	

TPIADL form

Task	Time	Score
1. **Pictorial-based communication task**: finding telephone numbers		☐ (1) completed without error and within 30 seconds<br>☐ (2) erroneous response within the time limit but corrected after a verbal cue<br>☐ (3) not completed with errors within the time limit and unable to correct even following a verbal cue
2. **Finance task**: finding and counting coins		☐ (1) completed without error and within 30 seconds<br>☐ (2) erroneous response within the time limit but corrected after a verbal cue<br>☐ (3) not completed with errors within the time limit and unable to correct even following a verbal cue
3. **Cooking task** : finding and reading the ingredients present in food stimulants		☐ (1) completed without error and within 30 seconds<br>☐ (2) erroneous response within the time limit but corrected after a verbal cue<br>☐ (3) not completed with errors within the time limit and unable to correct even following a verbal cue
4. **Shopping task**: finding two specified food items in an array of food items on a shelf		☐ (1) completed without error and within 30 seconds<br>☐ (2) erroneous response within the time limit but corrected after a verbal cue<br>☐ (3) not completed with errors within the time limit and unable to correct even following a verbal cue
5. **Pictorial-based medicine use task**: reading the directions on a medicine container		☐ (1) completed without error and within 30 seconds<br>☐ (2) erroneous response within the time limit but corrected after a verbal cue<br>☐ (3) not completed with errors within the time limit and unable to correct even following a verbal cue
	**Total score**	
